# TRI-PONDERAL MASS INDEX IS USEFUL FOR SCREENING CHILDREN AND ADOLESCENTS WITH INSULIN RESISTANCE

**DOI:** 10.1590/1984-0462/2020/38/2019066

**Published:** 2020-03-16

**Authors:** Felipe Silva Neves, Rafael de Oliveira Alvim, Divanei Zaniqueli, Virgilia Oliveira Pani, Caroline Resende Martins, Marcos Alves de Souza Peçanha, Míriam Carmo Rodrigues Barbosa, Eliane Rodrigues de Faria, José Geraldo Mill

**Affiliations:** a Universidade Federal de Juiz de Fora, Juiz de Fora, MG, Brazil.; b Universidade Federal do Amazonas, Manaus, AM, Brazil.; c Universidade Federal do Espírito Santo, Vitória, ES, Brazil.

**Keywords:** Child, Adolescent, Insulin resistance, Anthropometry, Body mass index, Tri-ponderal mass index, Criança, Adolescente, Resistência à insulina, Antropometria, Índice de massa corporal, Índice de massa tri-ponderal

## Abstract

**Objective:**

To investigate whether tri-ponderal mass index and body mass index Z scores are equivalent for screening children and adolescents with insulin resistance.

**Methods:**

Cross-sectional study with 296 children and adolescents enrolled at public schools of Vitória, Espírito Santo, Brazil, aged eight to 14 years. The tri-ponderal mass index was calculated as the ratio between weight and height cubed. The body mass index was calculated as the ratio between weight and height squared. Insulin resistance was defined with the homeostatic model assessment (HOMA-IR).

**Results:**

The HOMA-IR was higher in the 4^th^ quartile of body mass index Z scores and tri-ponderal mass index compared to 1^st^ and 2^nd^ quartiles for both girls and boys. The areas under the age-adjusted receiver operating characteristic curves were similar between the indices for girls (body mass index Z scores=0.756; tri-ponderal mass index=0.763) and boys (body mass index Z scores=0.831; tri-ponderal mass index=0.843). In addition, according to the simple linear regression analyses estimations, both body mass index Z scores and tri-ponderal mass index explained a significant fraction of the homeostatic model assessment variability for girls (body mass index Z scores: R^2^=0.269; tri-ponderal mass index: R^2^=0.289; p<0.001) and boys (body mass index Z scores: R^2^=0.175; tri-ponderal mass index: R^2^=0.210; p<0.001).

**Conclusions:**

The tri-ponderal mass index and body mass index Z scores were similar to discriminate children and adolescents with insulin resistance. It is noteworthy that the use of tri-ponderal mass index is clearly advantageous, because it can be calculated with no concerns on adjustments for the age, a fact that makes it very applicable in the clinical practice.

## RESUMO

**Objetivo:**

Investigar se o índice de massa tri-ponderal e os escores-z do índice de massa corporal são equivalentes na triagem de crianças e adolescentes com resistência à insulina.

**Métodos:**

Estudo transversal com 296 crianças e adolescentes matriculados em escolas públicas de Vitória, Espírito Santo, Brasil, com idades entre oito e 14 anos. O índice de massa tri-ponderal foi calculado como a razão entre o peso e a altura ao cubo. O índice de massa corporal foi calculado como a razão entre o peso e a altura ao quadrado. A resistência à insulina foi definida pelo modelo de avaliação da homeostase para resistência à insulina (HOMA-IR).

**Resultados:**

O HOMA-IR foi maior no 4^º^ quartil dos escores-z do índice de massa corporal e do índice de massa tri-ponderal, em relação ao 1^º^ e 2^º^ quartis em ambos os sexos. As áreas sob as curvas *receiver operating characteristic* ajustadas por idade foram semelhantes em meninas (escores-z do índice de massa corporal=0,756; índice de massa tri-ponderal=0,763) e meninos (escores-z do índice de massa corporal=0,831; índice de massa tri-ponderal=0,843). Além disso, de acordo com as estimativas das análises de regressão linear simples, os escores-z do índice de massa corporal e o índice de massa tri-ponderal explicaram uma fração significativa da variabilidade do modelo de avaliação da homeostase para meninas (escores-z do índice de massa corporal: R^2^=0,269; índice de massa tri-ponderal: R^2^=0,289; p<0,001) e meninos (escores-z do índice de massa corporal: R^2^=0,175; índice de massa tri-ponderal: R^2^=0,210; p<0,001).

**Conclusões:**

O índice de massa tri-ponderal e os escores-z do índice de massa corporal foram semelhantes na triagem de crianças e adolescentes com resistência à insulina. É importante ressaltar que o uso do índice de massa tri-ponderal é claramente vantajoso, pois pode ser calculado sem ajustes por idade, fato que o torna muito aplicável à prática clínica.

## INTRODUCTION

Insulin resistance (IR) is a metabolic disorder largely associated with obesity and it is recognized as a determining condition for the onset of type 2 diabetes mellitus (T2D).^1^ Among children, studies have shown that IR is significantly associated with obesity and cardiometabolic risk.^2^ Thus, the concern about alarming rates of T2D has motivated researchers to drive attention on the early IR onset and its close relationship with obesity.^3^ ^,^ ^4^ ^,^ ^5^

Due to the epidemic of childhood obesity,^6^ ^,^ ^7^ ^,^ ^8^ it is important to disseminate the necessity of early diagnosis of IR. The gold-standard technique for the IR assessment is the hyper-insulinemic-euglycemic clamp, elegantly developed by DeFronzo et al.^9^ However, despite the advantages of the hyper-insulinemic-euglycemic clamp over the indirect indices, it is an expensive, invasive and longstanding technique to be used in clinical settings and in epidemiologic studies, mainly involving pediatric populations. Therefore, simple and inexpensive surrogate indices, such as the homeostasis model assessment of insulin resistance (HOMA-IR) have been proposed. HOMA-IR has been shown as highly reliable as an estimator of IR in obese children and adolescents.^10^

The World Health Organization has proposed the use of body mass index Z scores (BMIz) as the most appropriate indicator to provide screening for pediatric obesity. However, a recent investigation has suggested that tri-ponderal mass index (TMI), a simple tool that does not involve complicated percentiles, calculated as weight divided by cubed height, achieved greater accuracy than BMIz in classifying overweight children and adolescents accordingly.^11^

Considering the critical role of obesity in triggering IR and the need for low cost and reliable tools to be used in a clinical setting, this study sought to investigate whether TMI and BMIz are equivalent in the screening of children and adolescents with IR.

## METHOD

The eligible sample for this cross-sectional study came from nine public schools of the municipality of Vitória, Espírito Santo, which is a city located in the Southeast of Brazil. Exclusion criteria were defined as: chronic or prolonged use of drugs that alter the metabolism of carbohydrates and lipids; declaration of chronic noncommunicable diseases or other diseases that promote inflammatory changes; statement of using pacemakers or orthopedic prostheses that compromise anthropometric and body composition assessments; people with special needs; and girls who reported gestation or lactation. A total of 296 children and adolescents of both sexes aged eight to 14 years were involved in the study. The sample size calculated to detect a difference of 0.50 in the value of HOMA-IR with a 5% error and 90% power was 269. From July 2016 to February 2017, the students attended the Cardiovascular Investigation Clinic located at the University Hospital, where they underwent clinical and laboratory examinations. Trained investigators previously certified by a senior investigator collected all data in a single visit.

The project was approved by the Institutional Ethics Committee (CAEE: 53609716.0.0000.5060; protocol: 1.565.490) and has been conducted in accordance with the Ethics Code of the World Medical Association (Declaration of Helsinki) for studies in humans. Written informed consent was obtained from parents or legal guardians before enrollment.

Anthropometric parameters were measured according to a standard protocol.^12^ Weight was obtained at the nearest 50 g with a calibrated electronic scale (Toledo, Brazil) on barefoot individuals that were only wearing underclothes. Height was obtained at the nearest 0.1 cm with a wall-mounted scale (Seca Stadiometer − Seca GmBH & Co, Hamburg, Germany). Percentage of body fat was measured by multi-frequency bioelectrical impedance analysis (MF-BIA8, InBody 230, Biospace, South Korea). The BMI was calculated as the ratio between weight and squared height (kg/m^2^). BMI for age and sex percentile was calculated according to the standards provided by the World Health Organization and further Z scores transformed.^13^ TMI was calculated through weight divided by cubed height (kg/m^3^).

Blood collection was obtained by venipuncture after overnight fasting (8 to 12 hours) and sent to a central laboratory (Laboratório Tommasi, Vitória, ES, Brazil) to determine serum concentrations of total cholesterol (TC), low density lipoprotein cholesterol (LDL-C), high density lipoprotein cholesterol (HDL-C), triglycerides (TG), glucose, and insulin. LDL-C was calculated by the Friedwald’s equation for those with TG ≤400 mg/dL.

HOMA-IR index was defined according to: [fasting insulin (U/mL) × fasting glucose (mmol/L) / 22.5].^14^ The presence of IR was set as HOMA-IR ≥3.16.^10^

Continuous data are expressed as mean and standard deviation, and categorical variables are expressed as number and percentages. The unpaired Student’s t-test was applied to compare the means of continuous variables between girls and boys. The comparison of proportions between girls and boys was conducted with the chi-square test.

BMIz and TMI were categorized into quartiles. Mean values of percentage of body fat, glucose, fasting insulin and HOMA-IR were compared between the quartiles through one-way ANOVA followed by Bonferroni post-hoc in case of a significant F test.

Receiver operating characteristic (ROC) curves were drawn to determine the discriminatory power of BMIz and TMI for IR, according to the cutoff value for HOMA-IR (≥3.16). The Epi package of the R-software 3.4.1 (http://www.r-project.org) was used to provide adjustment for age.

Simple linear regression analyses were performed for both BMIz and TMI as independent variables and HOMA-IR as a dependent variable. All analyses were performed separately for girls and boys.

Statistical procedures were carried out with *Statistical Package for the Social Sciences* (SPSS) 24.0 statistical package (SPSS Inc., Chicago, Illinois, USA) and GraphPad Prism 6.0 (GraphPad Software, Inc., CA, USA). Statistical significance was set at p<0.05.

## RESULTS

The main characteristics of the sample are exhibited in Table 1. Percentages of body fat (p<0.001), TG (p=0.005), fasting insulin (p=0.004), HOMA-IR (p=0.010), and proportion of insulin resistant individuals (p=0.005) were significantly higher in girls compared with boys.

**Table 1:** General characteristics of the sample.

Characteristics	All	Girls	Boys	p-value
n	296	161	135	-
Age (years)	10.2±3.3	10.2±3.3	10.2±3.1	0.922
Weight (kg)	39.2±19.7	40.6±19.8	38.4±19.7	0.867
Height (cm)	144.0±0.2	144.0±0.2	143.0±0.2	0.457
BMIz	0.68±1.41	0.65±1.41	0.72±1.43	0.681
TMI (kg/m^3^)	12.8±3.5	12.8±3.9	12.6±3.1	0.169
Body fat (%)	23.5±15.5	25.5±15.3	21.3±13.8	<0.001
TC (mg/dL)	149.0±31.7	148.0±29.5	149.0±38.0	0.760
LDL-C (mg/dL)	77.5±27.0	76.0±25.0	78.0±30.0	0.527
HDL-C (mg/dL)	51.0±13.7	50.0±14.0	52.0±14.0	0.051
TG (mg/dL)	67.0±37.0	73.0±47.0	63.0±33.0	0.005
Glucose (mg/dL)	87.0±10.7	87.0±11.0	88.0±10.0	0.244
Insulin (mcUI/mL)	10.6±8.2	11.9±9.0	9.8±7.4	0.004
HOMA-IR	2.27±1.82	2.49±2.02	2.02±1.68	0.010
IR (%)	78 (26.4)	53 (32.9)	25 (18.5)	0.005

BMIz: body mass index Z scores; TMI: tri-ponderal mass index; TC: total cholesterol; LDL-C: low-density lipoprotein cholesterol; HDL-C: high-density lipoprotein cholesterol; TG: triglycerides; HOMA-IR: homeostasis model assessment-insulin resistance; IR: insulin resistance; values are mean±standard deviation or number and percentage; unpaired Student’s t-test or chi-square test; significant difference at p-value<0.05.

Both indices were accurate in estimating body fat percentage (Table 2). A significantly higher body fat percentage was observed among girls and boys in the upper quartiles for BMIz and TMI. For them, the fasting glucose level was similar between the quartiles. Fasting insulin and HOMA-IR, however, were higher in the 4^th^ quartile of BMIz compared to the 1^st^, 2^nd^ and 3^rd^ quartiles for both genders.

**Table 2:** Body fat and fasting glucose metabolism variables according to quartiles of body mass index Z scores and tri-ponderal mass index.

	BMIz				TMI			
	1^st^ quartile	2^nd^ quartile	3^rd^ quartile	4^th^ quartile	1^st^ quartile	2^nd^ quartile	3^rd^ quartile	4^th^ quartile
Girls								
n	40	41	40	40	45	37	42	37
Body fat (%)	17.1±2.3	23.1±1.5	30.7±3.2	41.6±3.7*	17.5±2.5	23.4 ± 1.3	31.2±3.3	42.0±3.6*
Glucose (mg/dL)	85.6±6.6	86.4±8.0	86.3±10.4	87.8±7.6	85.4±6.5	87.0±8.2	86.4±10.2	87.6±7.9
Insulin (mcUI/mL)	8.2±4.5	11.0±5.6	14.8±6.2	19.6±10.5*	8.4±4.4	11.2±5.8	14.7±6.4	20.2±10.5*
HOMA-IR	1.7±1.0	2.4±1.3	3.2±1.5	4.4±2.6*	1.8±0.9	2.4±1.4	3.2±1.5	4.5±2.6*
Boys								
n	35	33	34	33	33	36	33	33
Body fat (%)	12.4±1.8	17.7±2.0	24.9±2.3	35.7±6.1*	12.5±1.7	17.8±2.1	25.2±2.3	35.1±7.7*
Glucose (mg/dL)	88.7±7.7	87.5±9.0	87.0±10.5	87.5±7.9	89.2±7.6	85.8±11.4	89.0±7.3	86.9±7.7
Insulin (mcUI/mL)	7.5±3.5	9.3±4.9	10.3±4.9	15.9±11.0*	7.4±3.5	9.2±4.8	11.2±6.6	15.1±10.4**
HOMA-IR	1.6±0.8	2.0±1.1	2.3±1.2	3.5±2.6*	1.6±0.8	2.0±1.1	2.5±1.6	3.3±2.4**

BMIz: body mass index Z scores; TMI: tri-ponderal mass index; HOMA-IR: homeostasis model assessment-insulin resistance; values are the mean ± standard deviation; one-way ANOVA; significant difference at p-value <0.05; *different from 1^st^, 2^nd^, and 3^rd^ quartiles; **different from 1^st^ and 2^nd^ quartiles.

**Figure 1:**
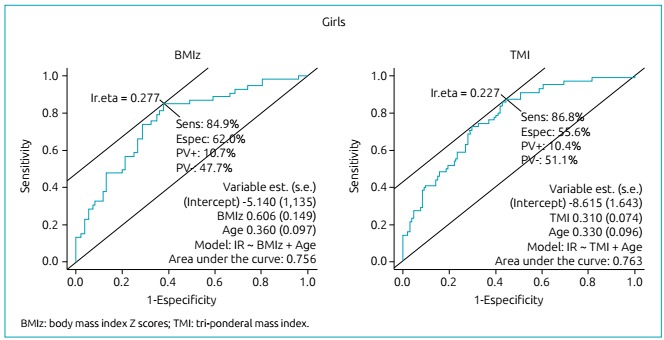
Discriminatory power of anthropometric indicators, such as body mass index Z scores and tri-ponderal mass index for screening girls with insulin resistance.

**Figure 2:**
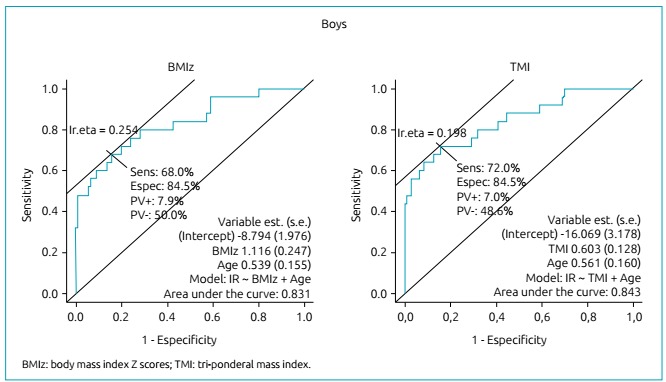
Discriminatory power of anthropometric indicators, such as body mass index Z scores and tri-ponderal mass index for screening boys with insulin resistance.

ROC curves adjusted for age were drawn to test the ability of BMIz and TMI to correctly classify those individuals classified as insulin resistant. The areas under the ROC curves were quite similar between BMIz and TMI for girls (0.756 *versus* 0.763, respectively) and for boys (0.831 *versus* 0.843, respectively), as seen in Figures 1 and 2.

**Figure 3:**
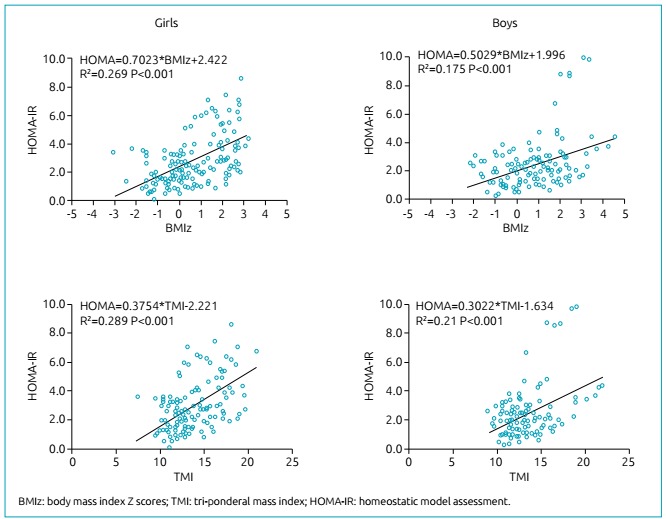
Simple linear regression for both body mass index Z scores (parts A and B) and tri-ponderal mass index (parts C and D) as independent variables with the homeostasis model assessment for insulin resistance.

Scatter plots show the linear regression of HOMA-IR on BMIz and TMI (Figure 3). A significant linear increase in the HOMA-IR value with increasing BMIz was observed for girls (β=0.70/unit) and boys (β=0.50/unit) (both with p<0.001). Accordingly, BMIz explained 26.9 and 17.5% of the variability of HOMA-IR for girls and boys, respectively (Figure 3, parts A and B). A significant linear increase in the HOMA-IR value with increasing TMI was observed for girls (β=0.38/unit) and boys (β=0.30/unit) (both with p<0.001). Of the total variability of HOMA-IR, 28.9 and 21.0% can be explained by the TMI variability for girls and boys, respectively (Figure 3, parts C and D).

## DISCUSSION

The results of this study confirmed that TMI was similar to BMIz in the identification of IR in children and adolescents. In addition, both anthropometric indicators presented similar discriminatory power for IR.

The ponderal index (same as TMI) has been used to assess neonates’ body proportionality, as it allows a differentiation between symmetric and asymmetric growth restriction.^15^ Previous studies have demonstrated that thinness (*i.e.* low TMI) at birth is a predictor for IR during adulthood.^5^ ^,^ ^16^ ^,^ ^17^ Although 0‒2 years has been considered the limit age group for using TMI,^18^ recently TMI has been seen as more accurate than BMI Z scores in estimating body fat in a population consisting of children and young adults (8‒29 years).^11^

To our knowledge, no study has tested the TMI as a predictor of IR in pediatric populations. In contrast, BMIz has been extensively used to predict IR in pre-pubertal children ^19^ ^,^ ^20^ and adolescents.^21^ ^,^ ^22^

In the present study, TMI and BMIz were similar to discriminate IR and to predict HOMA-IR. As TMI and BMIz increase, the upward shift in HOMA-IR value seems to be steeper in girls than in boys, though this comparison is out of this study scope.

Several mechanisms have been proposed as possibilities to explain the association between adiposity and IR. Hypertrophy and hyperplasia of adipocytes are common adaptations of positive energetic balance, which consequently increase the oxidative stress. It, in turn, increases the production of adipokines and inflammatory mediators, which are associated with peripheral and hepatic IR, and impaired insulin secretion by pancreatic beta cells.^23^ A growing body of evidence, however, has provided support to the role of body fat distribution (subcutaneous truncal and abdominal) on the underlying mechanisms of IR.^24^ ^,^ ^25^ Therefore, it is a matter of concern whether BMI is a reasonable index to estimate these adipose tissue depots.

An elegant study^26^ questioned why weight scales to height with a power of two (express area) and not with a power of three (express volume) in the calculus of BMI. The author argued that for objects with different sizes (*i.e.* length in humans) but identical shape (a single cylinder as in humans), the volume has to proportional to the cube of length, and the ratio mass/length^3^ tends to be constant. Moreover, even with variations in shape, mass/(length^3^) continues to be a full index of shape, regardless of the size.^26^ Conversely, BMI, even if Z scores are transformed, can be biased in pediatric populations since the attainment of height is associated with fatness, because boys and girls with obesity tend to be taller than their lean age-matched counterparts.^27^ ^,^ ^28^

Recently, our group reproduced the protocol by Peterson et al.^11^ Our analyses indicated that TMI was superior to BMIz to appropriately diagnose obesity. However, both indices had similar accuracy in estimating percentage of body fat.^29^ Thus, we have reasons to believe that TMI and BMIz presented similar discriminatory power for pediatric IR, probably because both indices are equivalent in estimating the fat deposits that are directly associated with insulin resistance. Even so, we strongly recommend the use of TMI by clinicians rather than BMIz, since it is very stable from childhood to youth,^11^ ^,^ ^29^ besides being free of complicate percentile tables.

The identification of useful, convenient and economic screening tools to detect IR during childhood and early adulthood is of particular interest, since this will facilitate more timely and effective interventions in those subjects who are at greater risk. Hence, TMI clearly shows some advantages over BMIz, because it can be calculated with no concerns on adjustments for age and is more accurate in screening for pediatric obesity.^11^ ^,^ ^29^ In addition, unlike biochemical markers, which always require some cost, besides the need of fasting blood collection, TMI can be used in the absence of invasive laboratory exams, provides instant results and is free of costs. These characteristics make TMI easier to be used by both healthcare providers and children and adolescents themselves. This information is very relevant to the public health. However, since most clinicians and researchers utilize BMIz, there would have to be an overall proven increase in usefulness of TMI in order to convince the healthcare community to adopt this alternate measure.

Despite being the first study to address the discrimination ability of TMI for IR in children and adolescents, some limitations need to be considered. First, the sample was not randomly assigned; therefore, the results cannot be extrapolated to the general population with a similar age range. Secondly, there is a high miscegenation in the Brazilian population and therefore more population-based studies conducted in other ethnic or racial groups are required. Thirdly, despite HOMA-IR has been widely used in the clinical practice for IR diagnosis, it would be important to assess the discriminatory power of the indicators using the hyper-insulinemic-euglycemic clamp gold standard as reference.

In summary, TMI was equivalent to BMIz to discriminate children and adolescents with IR. Therefore, it is plausible that the combined use of both indices be encouraged. The use of TMI is clearly advantageous, considering this index can be calculated without adjustments for age, which makes it very applicable in the clinical practice.

## References

[1] Popkin BM, Adair LS, Ng SW (2012). Global nutrition transition and the pandemic of obesity in
developing countries. Nutr Rev.

[2] Ten S, Maclaren N (2004). Insulin resistance syndrome in children. J Clin Endocrinol Metab.

[3] Sinha R, Fisch G, Teague B, Tamborlane WV, Banyas B, Allen K (2002). Prevalence of impaired glucose tolerance among children and
adolescents with marked obesity. N Engl J Med.

[4] Santiago-Torres M, Cui Y, Adams AK, Allen DB, Carrel AL, Guo JY (2016). Familial and individual predictors of obesity and insulin
resistance in urban Hispanic children. Pediatr Obes.

[5] Nogueira-de-Almeida CA, Mello ED (2018). Correlation of BMI Z-scores with glucose and lipid profiles among
overweight and obese children and adolescents. J Pediat (Rio J).

[6] Ng M, Fleming T, Robinson M, Thomson B, Graetz N, Margono C (2014). Global, regional, and national prevalence of overweight and
obesity in children and adults during 1980-2013: a systematic analysis for
the global burden of disease study 2013. Lancet.

[7] World Health Organization (2016). Consideration of the evidence on childhood obesity for the Commission on
Ending Childhood Obesity: report of the ad hoc working group on science and
evidence for ending childhood obesity.

[8] NCD Risk Factor Collaboration (2017). Worldwide trends in body mass index, underweight, overweight, and
obesity from 1975 to 2016: a pooled analysis of 2416 population-based
measurement studies in 128.9 million children, adolescents, and
adults. Lancet.

[9] DeFronzo RA, Tobin JD, Andres R. (1979). Glucose clamp technique: a method for quantifying insulin
secretion and resistance. Am J Physiol.

[10] Keskin M, Kurtoglu S, Kendirci M, Atabek ME, Yazici C (2005). Homeostasis model assessment is more reliable than the fasting
glucose/insulin ratio and quantitative insulin sensitivity check index for
assessing insulin resistance among obese children and
adolescents. Pediatrics.

[11] Peterson CM, Su H, Thomas DM, Heo M, Golnabi AH, Pietrobelli A (2017). Tri-ponderal mass index vs body mass index in estimating body fat
during adolescence. JAMA Pediatr.

[12] Lohman T, Roche A, Martorell E (1988). Anthropometric standardization reference manual.

[13] de Onis M, Onyango AW, Borghi E, Siyam A, Nishida C, Siekmann J (2007). Development of a WHO growth reference for school-aged children
and adolescents. Bull World Health Organ.

[14] Matthews DR, Hosker JP, Rudenski AS, Naylor BA, Treacher DF, Turner RC (1985). Homeostasis model assessment: insulin resistance and beta-cell
function from fasting plasma glucose and insulin concentrations in
man. Diabetologia.

[15] Landmann E, Reiss I, Misselwitz B, Gortner L (2006). Ponderal index for discrimination between symmetric and
asymmetric growth restriction: percentiles for neonates from 30 weeks to 43
weeks of gestation. J Matern Fetal Neonatal Med.

[16] Phillips DI, Barker DJ, Hales CN, Hirst S, Osmond C (1994). Thinness at birth and insulin resistance in adult
life. Diabetologia.

[17] Thompson CH, Sanderson AL, Sandeman D, Stein C, Borthwick A, Radda GK (1997). Fetal growth and insulin resistance in adult life: role of
skeletal muscle morphology. Clin Sci (Lond).

[18] Howe LD, Tilling K, Benfield L, Logue J, Sattar N, Ness AR (2010). Changes in ponderal index and body mass index across childhood
and their associations with fat mass and cardiovascular risk factors at age
15. PLoS One.

[19] Bennett B, Larson-Meyer DE, Ravussin E, Volaufova J, Soros A, Cefalu WT (2012). Impaired insulin sensitivity and elevated ectopic fat in healthy
obese vs. nonobese prepubertal children. Obesity (Silver Spring).

[20] Mueller NT, Pereira MA, Buitrago-Lopez A, Rodríguez DC, Duran AE, Ruiz AJ (2013). Adiposity indices in the prediction of insulin resistance in
prepubertal Colombian children. Public Health Nutr.

[21] Burrows RA, Leiva LB, Weisstaub G, Lera LM, Albala CB, Blanco E (2011). High HOMA-IR, adjusted for puberty, relates to the metabolic
syndrome in overweight and obese Chilean youths. Pediatr Diabetes.

[22] Gobel RJ, Jensen SM, Frøkiaer H, Mølgaard C, Michaelsen KF (2012). Obesity, inflammation and metabolic syndrome in Danish
adolescents. Acta Paediatr.

[23] Aa MP, Farsani SF, Knibbe CA, Boer A, Vorst MM (2015). Population-based studies on the epidemiology of insulin
resistance in children. J Diabetes Res.

[24] Patel P, Abate N (2013). Role of subcutaneous adipose tissue in the pathogenesis of
insulin resistance. J Obes.

[25] Patel P, Abate N (2013). Body fat distribution and insulin resistance. Nutrients.

[26] Burton RF (2007). Why is the body mass index calculated as mass/height2, not as
mass/height3?. Ann Hum Biol.

[27] Garn SM, Haskell JA (1960). Fat thickness and developmental status in childhood and
adolescence. AMA J Dis Child.

[28] Garn SM, Clark DC, Guire KE (1974). Levels of fatness and size attainment. Am J Phys Anthropol.

[29] Zaniqueli D, Oliosa PR, Neves FS, Pani VO, Martins CR, Peçanha MA (2019). Ponderal index classifies obesity in children and adolescents
more accurately than body mass index z-scores. Pediatr Res.

